# Importance of Poly-3-Hydroxybutyrate Metabolism to the Ability of Herbaspirillum seropedicae To Promote Plant Growth

**DOI:** 10.1128/AEM.02586-18

**Published:** 2019-03-06

**Authors:** Luis Paulo Silveira Alves, Fernanda Plucani do Amaral, Daewon Kim, Maritza Todo Bom, Manuel Piñero Gavídia, Cícero Silvano Teixeira, Fernanda Holthman, Fabio de Oliveira Pedrosa, Emanuel Maltempi de Souza, Leda Satie Chubatsu, Marcelo Müller-Santos, Gary Stacey

**Affiliations:** a Biological Nitrogen Fixation Laboratory, Department of Biochemistry and Molecular Biology, Federal University of Paraná (UFPR), Curitiba, Brazil; b Divisions of Plant Science and Biochemistry, C. S. Bond Life Science Center, University of Missouri, Columbia, Missouri, USA; Goethe University Frankfurt am Main

**Keywords:** *Herbaspirillum seropedicae*, PHB, *Setaria viridis*, plant growth promotion, polyhydroxybutyrate, rhizosphere

## Abstract

Herbaspirillum seropedicae is an endophytic bacterium that establishes an association with a variety of plants, such as rice, corn, and sugarcane, and can significantly increase plant growth. H. seropedicae produces polyhydroxybutyrate (PHB), stored in the form of insoluble granules. Little information is available on the possible role of PHB in bacterial root colonization or in plant growth promotion. To investigate whether PHB is important for the association of H. seropedicae with plants, we inoculated roots of Setaria viridis with H. seropedicae strain SmR1 and mutants defective in PHB production (Δ*phaP1*, Δ*phaP1* Δ*phaP2*, Δ*phaC1*, and Δ*phaR*) or mobilization (Δ*phaZ1* Δ*phaZ2*). The strains producing large amounts of PHB colonized roots, significantly increasing root area and the number of lateral roots compared to those of PHB-negative strains. H. seropedicae grows under microaerobic conditions, which can be found in the rhizosphere. When grown under low-oxygen conditions, only the parental strain and Δ*phaP2* mutant exhibited normal growth. The lack of normal growth under low oxygen correlated with the inability to stimulate plant growth, although there was no effect on the level of root colonization. The data suggest that PHB is produced in the root rhizosphere and plays a role in maintaining normal metabolism under microaerobic conditions. To confirm this, we screened for green fluorescent protein (GFP) expression under the control of the H. seropedicae promoters of the PHA synthase and PHA depolymerase genes in the rhizosphere. PHB synthesis is active on the root surface and later PHB depolymerase expression is activated.

**IMPORTANCE** The application of bacteria as plant growth promoters is a sustainable alternative to mitigate the use of chemical fertilization in agriculture, reducing negative economic and environmental impacts. Several plant growth-promoting bacteria synthesize and accumulate the intracellular polymer polyhydroxybutyrate (PHB). However, the role of PHB in plant-bacterium interactions is poorly understood. In this study, applying the C4 model grass Setaria viridis and several mutants in the PHB metabolism of the endophyte Herbaspirillum seropedicae yielded new findings on the importance of PHB for bacterial colonization of S. viridis roots. Taken together, the results show that deletion of genes involved in the synthesis and degradation of PHB reduced the ability of the bacteria to enhance plant growth but with little effect on overall root colonization. The data suggest that PHB metabolism likely plays an important role in supporting specific metabolic routes utilized by the bacteria to stimulate plant growth.

## Abstract (precis)

The application of bacteria as plant growth promoters is a sustainable alternative to mitigate the use of chemical fertilization in agriculture, reducing negative economic and environmental impacts. Several plant growth-promoting bacteria synthesize and accumulate the intracellular polymer polyhydroxybutyrate (PHB). However, the role of PHB in plant-bacterium interactions is poorly understood. In this study, applying the C4 model grass Setaria viridis and several mutants in the PHB metabolism of the endophyte Herbaspirillum seropedicae yielded new findings on the importance of PHB for bacterial colonization of S. viridis roots. Taken together, the results show that deletion of genes involved in the synthesis and degradation of PHB reduced the ability of the bacteria to enhance plant growth but with little effect on overall root colonization. The data suggest that PHB metabolism likely plays an important role in supporting specific metabolic routes utilized by the bacteria to stimulate plant growth.

## INTRODUCTION

Several bacteria accumulate polymers which can serve as a source of energy and carbon storage, especially under stressful or limited-nutrient conditions (1, 2). Polyhydroxyalkanoates (PHA) are polymers often produced and accumulated by several species of bacteria (3). Poly-3-hydroxybutyrate (PHB; also referred to as polyhydroxybutyrate) is the PHA typically produced by prokaryotes (4). PHB is an aliphatic polyester synthesized in three steps: (i) acetyl coenzyme A (acetyl-CoA) condensation generating acetoacetyl-CoA by the beta-ketothiolase PhaA, (ii) reduction of acetoacetyl-CoA into 3-hydroxybutyryl-CoA by the NADPH-dependent acetoacetyl-CoA reductase PhaB, and (iii) 3-hydroxybutyryl-CoA condensation in PHB by the PHA synthase PhaC. The PHB is insoluble in water and consequently accumulates within the cytoplasm. The PHB inside the bacteria is amorphous and coated by structural proteins, forming PHB granules (5). Phasins are the most abundant proteins attached to the surface of PHB granules, regulating the size and the number of PHB granules inside the cell (6–9). The negative regulator PhaR represses the transcription of *phaP* (encoding the phasin PhaP) in several PHB-producing bacteria (10–14). When the bacterium synthesizes the initial chains of PHB, PhaR binds to the nascent PHB, releasing *phaP* repression (9, 15, 16). As granules grow and binding of the granule-associated proteins (GAPs) increases (17), there is no more surface for PhaR binding, leading to the binding of PhaR in the operator sites upstream of *phaP* and *phaR* genes.

Bacteria mobilize PHB granules through the hydrolytic activity of the PHA depolymerase PhaZ under certain environmental and nutritional conditions (18). The mechanisms controlling the PHB mobilization are not fully understood. However, some factors, such as the alarmone ppGpp (guanosine tetraphosphate) level (19, 20), protein synthesis rate and cellular energy demand (21), and oxidative stress (22), are possible triggers for PHB mobilization. Therefore, balance between synthesis and mobilization of PHB is an important metabolic cycle named the PHB cycle (23). The PHB cycle has been implicated as a beneficial feature to the survival of bacteria in the environment (24). Bacteria inhabiting competitive environments such as soil and the plant rhizosphere require an energy contribution to face unfavorable conditions of growth (25). In this sense, the functioning of the PHB cycle represents a beneficial feature for such organisms to compete and to recognize and colonize their hosts (26). The role of the PHB metabolism in plant-associated bacteria has been well studied in rhizobia and rhizobium-legume interactions (23). For instance, Sinorhizobium meliloti and Rhizobium leguminosarum accumulate large amounts of PHB in the rhizosphere, because it is a nutrient-rich environment. This PHB is subsequently mobilized in the bacteroids (symbiotic form) (27), providing carbon skeletons to synthetic and energetic metabolism, as well as providing reducing power for nitrogen fixation (28). In contrast to this work in rhizobia, less is known regarding the effects of the PHB cycle in other species of bacteria typically associated with grasses (26, 29, 30).

The plant growth-promoting, endophytic, and diazotrophic betaproteobacterium Herbaspirillum seropedicae has emerged as a model to investigate the processes of bacterial recognition, colonization, and growth promotion in grasses (31, 32). H. seropedicae can colonize a wide variety of grass species, including rice, sorghum, maize, and sugarcane (33–35). The genome of H. seropedicae strain SmR1 was sequenced, revealing the presence of at least 13 genes involved in the metabolism of PHB. The availability of the genome sequence enabled the construction of a variety of mutants showing defects in the regulation, synthesis, or degradation of PHB (13, 15, 36, 37).

In order to determine whether PHB synthesis and mobilization are relevant to H. seropedicae during root colonization or for the organism's ability to stimulate plant growth, we examined the ability of the parental H. seropedicae SmR1 strain and several mutants defective in PHB production (Δ*phaP1*, Δ*phaP1* Δ*phaP2*, Δ*phaC1*, and Δ*phaR*) or mobilization (Δ*phaZ1* Δ*phaZ2*) to colonize the roots of the model grass Setaria viridis A10.1, which we previously demonstrated showed a strong, positive growth response to inoculation (38).

## RESULTS

### The deletion of genes involved in the PHB metabolism impacts plant growth promotion by H. seropedicae SmR1.

**FIG 1:**
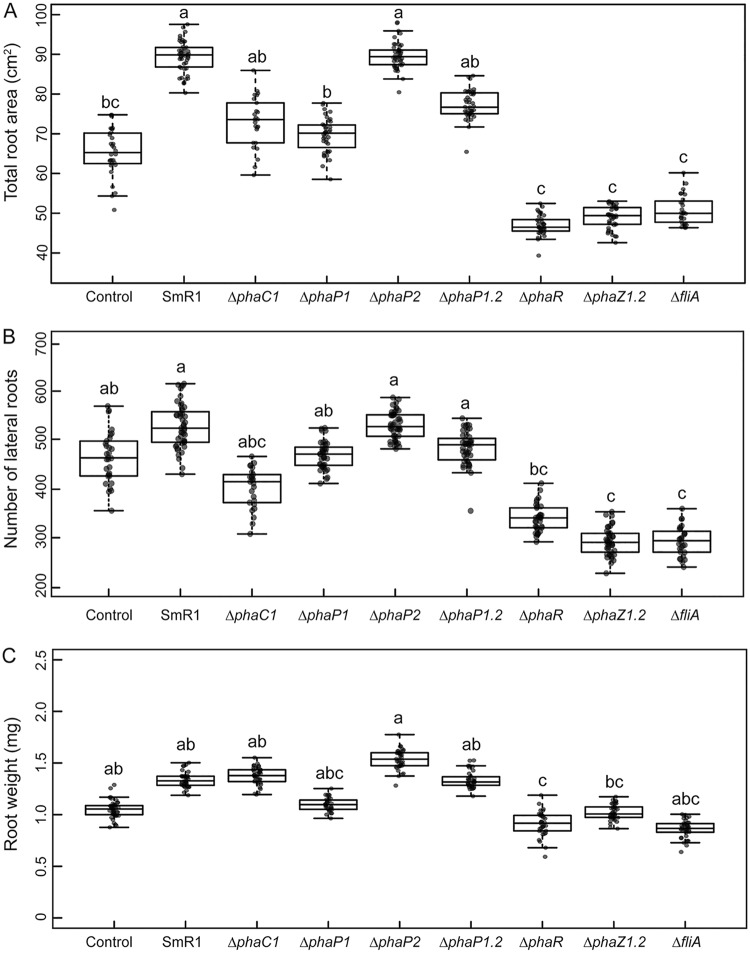
Effect of inoculation of S. viridis roots with H. seropedicae SmR1 and PHB defective mutants. (A) Analysis of total root area of *Setaria viridis* A10.1 after inoculation with various H. seropedicae strains. (B) Impact of bacterial inoculation on stimulation of lateral root formation. (C) Impact of bacterial inoculation on plant root weight. Plants were inoculated with H. seropedicae SmR1 (parental strain) and mutants as indicated below each box plot in the graph. The inoculated and uninoculated plants were grown in a potting mix composed of Turface and vermiculite. “Control” refers to uninoculated plants. The roots from plants grown 25 days after inoculation were washed and analyzed using WinRHIZO pro software (Regent Instruments, Canada). Box plots represent the measurement distribution, with individual values superimposed as dots. The horizontal line inside the box represents the mean, the box limits represent the 25th and 75th percentiles, and the whiskers represent the minimal and maximal values of the SE. Letters a to c indicate statistical distinct groups according to one-way ANOVA *post hoc* Tukey test (*P* < 0.05). The same letters between the groups indicate nonsignificant differences (*P* > 0.05) among the means. “Δ*phaP1.2*” represents the Δ*phaP1* Δ*phaP2* mutant.

**FIG 2:**
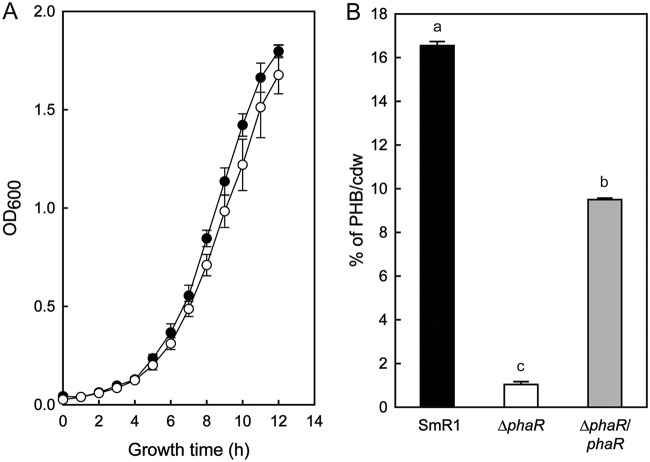
Growth and PHB accumulation profiles of H. seropedicae SmR1 and the Δ*phaR* mutant. (A) Bacteria were grown in NFbHPN-malate medium with 20 mM ammonium chloride and 37 mM dl-malate at 30°C and 120 rpm (orbital shaking). Bacterial growth was measured by OD_600_ from four independent cultures. Black circles represent SmR1, and white circles represent the Δ*phaR* mutant. (B) PHB contents were measured by whole-cell methanolysis and gas chromatography from three independent samples of each strain when they reached an OD_600_ of 1.0 cultivated under the same conditions as for panel A. The quantity of accumulated PHB was normalized by the cell dry weight (cdw) of lyophilized bacterial cultures used in the experiment. “Δ*phaR*/*phaR*” represents the mutant complemented with a plasmid-borne copy of *phaR* from H. seropedicae SmR1. Letters a to c indicate statistical distinct groups according to one-way ANOVA *post hoc* Tukey test (*P* < 0.05).

Previous research demonstrated a significant impact on fresh root weight, lateral root number, and total root area when *S. viridis* plants were inoculated with H. seropedicae and Azospirillum brasilense (38). To examine whether PHB metabolism is important during plant-bacterium interaction, we inoculated S. viridis A10.1 plants with H. seropedicae SmR1 and various mutants defective in PHB metabolism (13, 37). Consistent with our previous findings, the inoculation with H. seropedicae SmR1 increased the total root area 37% compared to that of the control (uninoculated plants) (Fig. 1A). However, the number of lateral roots and the root weight of SmR1 inoculated plants were not statistically different from those of the control (Fig. 1B and C). Plants inoculated with the Δ*phaC1* mutant, which does not produce PHB, had a 20% reduction in total root area and lateral root number compared to those of the parental SmR1. Plants inoculated with the Δ*phaP1* mutant, which produced 50% less PHB than strain SmR1 (13), showed a decrease of 23% in total root area and root dry weight (Fig. 1A and C). It is noteworthy that plants inoculated with the Δ*phaP1* Δ*phaP2* double mutant, lacking both phasins PhaP1 and PhaP2 and unable to store PHB in granules (13), showed a 12% reduction of total root area in comparison to that with SmR1 (Fig. 1A). On the other hand, plants inoculated with the Δ*phaP2* mutant, which produces wild-type levels of PHB, had growth properties similar to those of the SmR1-inoculated plants for the three measured parameters. Plants inoculated with the Δ*phaR* mutant, which produced only 1% PHB/cell (dry weight) compared to 16% PHB/cell (dry weight) in strain SmR1 (Fig. 2), had a remarkable reduction of 47% in the total root area and 26% in the number of lateral roots compared to those with SmR1 (Fig. 1A and B).

Our results suggest that PHB metabolism is required for the ability of H. seropedicae to fully promote the growth of S. viridis. It is interesting that not all the phenotypes measured were equally impacted, although the general trends were consistent.

### Disruption of PHB degradation also decreases bacterial plant growth promotion.

PHB mobilization is correlated with bacterial survival under high temperature, osmotic stress, UV radiation, and oxidative stress (20, 22, 39). The genome of H. seropedicae SmR1 encodes two putative PHA depolymerases, PhaZ1 (locus tag Hsero_1622) and PhaZ2 (locus tag Hsero_0639), as identified by amino acid sequence alignment against PhaZ1a1 (locus tag A1150; 66% identity) and PhaZ2a2 (locus tag A2862; 59% identity) from Ralstonia eutropha H16, respectively (see Fig. S1 in the supplemental material). Furthermore, PhaZ1 from H. seropedicae SmR1 was previously localized on the surface of PHB granules, in a position consistent with a role in degrading the insoluble polymer (36). Given that gene deletions that affect PHB biosynthesis affected plant growth promotion (Fig. 1), we tested whether the ability to mobilize the PHB reserve might also be essential. We inoculated S. viridis roots with an H. seropedicae Δ*phaZ1* Δ*phaZ2* double mutant, lacking both enzymes PhaZ1 and PhaZ2. Plant growth promotion by this mutant strain was severely impacted as measured by decreases of 44% in total root area (Fig. 1A), 45% in lateral root number (Fig. 1B), and 24% in root dry weight (Fig. 1C) compared to those of the parental strain SmR1.

### Low oxygen affects the growth of mutants producing less PHB.

**FIG 3:**
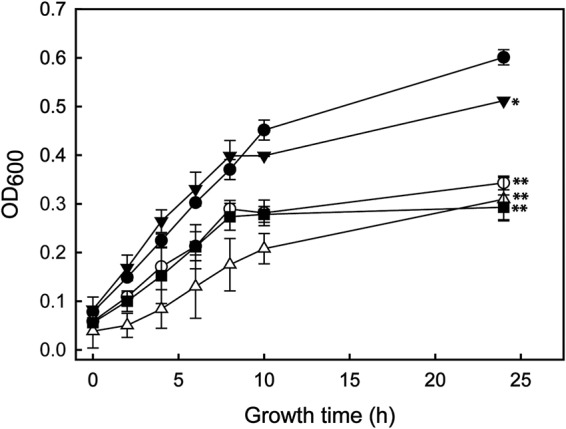
Growth profiles of H. seropedicae SmR1 (parental strain) and the Δ*phaP1*, Δ*phaP2*, Δ*phaP1* Δ*phaP2* (Δ*phaP1.2*), and Δ*phaC1* mutants under low-oxygen conditions. Strains were grown in NFbHPN-malate medium with 20 mM ammonium chloride and 37 mM dl-malate at 30°C and 100 rpm (orbital shaking) in glass flasks of 60-ml capacity filled with 50 ml of medium and closed with rubber caps. Results for SmR1 (black circles) and the Δ*phaP1* (white circles), Δ*phaP2* (black inverted triangles), Δ*phaP1.2* (white triangles), and Δ*phaC1* (black squares) mutants are shown. Bacterial growth was measured in function of the OD_600_ from three independent cultures. The whiskers represent the minimal and maximal values of the SEs. After 24 h of growth, the strain SmR1 showed statistical significance against the other strains employed in the experiment (*, *P* value ≤ 0.01; **, *P* value ≤ 0.001) by the one-way ANOVA, *post hoc* Tukey test (*P* < 0.05).

The level of oxygen affects the synthesis of PHB as shown previously for rhizobial bacteroids that produce PHB and lipids inside legume root nodules, a low-oxygen environment (40, 41). Also, a number of plant growth-promoting rhizobacteria (e.g., *Herbaspirillum*) are known to be microaerophilic (42). Based on this, we tested whether low-oxygen conditions could affect the growth of mutants defective in PHB synthesis or degradation. In order to simulate a low-oxygen condition, we grew SmR1 and mutant strains under a low-oxygen regimen as described in Materials and Methods. Those strains accumulating large amounts of PHB, such as SmR1 and the Δ*phaP2* mutant, showed faster growth than those mutants that produce less or no PHB (Δ*phaC1*, Δ*phaP1*, Δ*phaP1* Δ*phaP2*, and Δ*phaR* mutants) (Fig. 3).

### Defective PHB metabolism does not affect bacterial colonization of plant roots.

**FIG 4:**
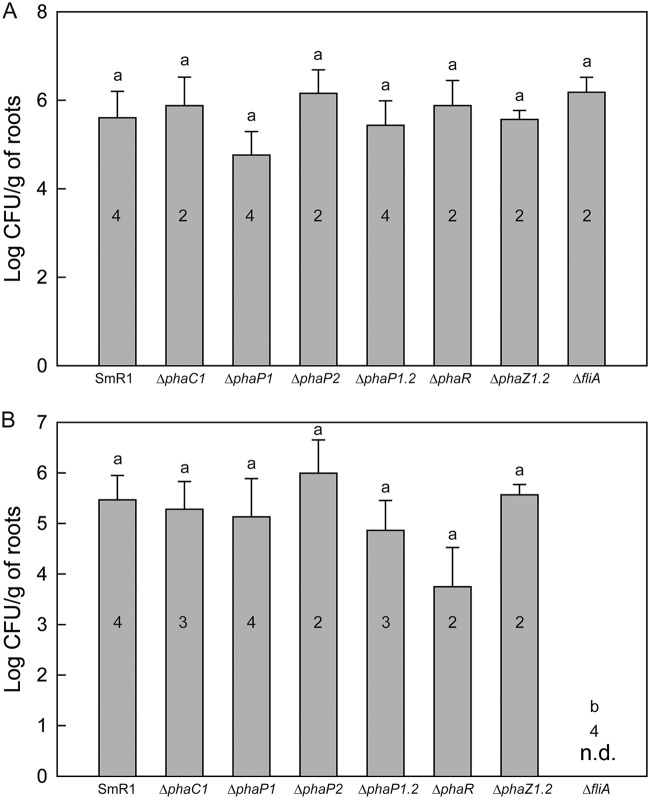
Bacterial colonization of roots of S. viridis A10.1 25 days after inoculation. Data are expressed in log CFU per gram of fresh roots. (A) Epiphytic root colonization was measured by counting the number of colonies of H. seropedicae recovered from external root tissue without sterilization. (B) Endophytic root colonization was measured by counting the number of colonies of H. seropedicae from macerated roots after surface sterilization. The numbers inside the bars indicate the number of plants analyzed in the experiment. Letters a and b indicate statistical distinct groups according to one-way ANOVA *post hoc* Tukey test (*P* < 0.05). The same letters between the groups indicate nonsignificant differences (*P* > 0.05) among the means. Note that there was no significant difference in colonization for the various strains tested, except for the endophytic colonization of the Δ*fliA* mutant, which was below detectable limits (n.d.).

The lack of effective plant growth promotion by H. seropedicae strains defective in PHB metabolism could be due to direct effects on bacterial colonization given the low-oxygen conditions that likely exist in the root rhizosphere (43). To examine this hypothesis, S. viridis A10.1 plants were inoculated with H. seropedicae SmR1 and mutant strains, and both epiphytic and endophytic colonization was measured by plate counting after 25 days. Regardless of which PHB mutant was introduced, there were no significant differences in root colonization (epiphytic or endophytic) when the bacteria were recovered from roots of S. viridis 25 days after inoculation (Fig. 4). These results are consistent with previous studies comparing root colonization in maize and wheat by wild-type A. brasilense Sp7 and the PHB-defective *phbC* mutant (29). Similar findings were also reported by Balsanelli et al. (44), who assayed colonization of maize roots and found no significant differences in root colonization between roots inoculated with H. seropedicae SmR1 and the Δ*phaC1* strain. Likewise, previous experiments indicate that total root colonization does not correlate with the level of bacterial plant growth promotion (38).

**FIG 5:**
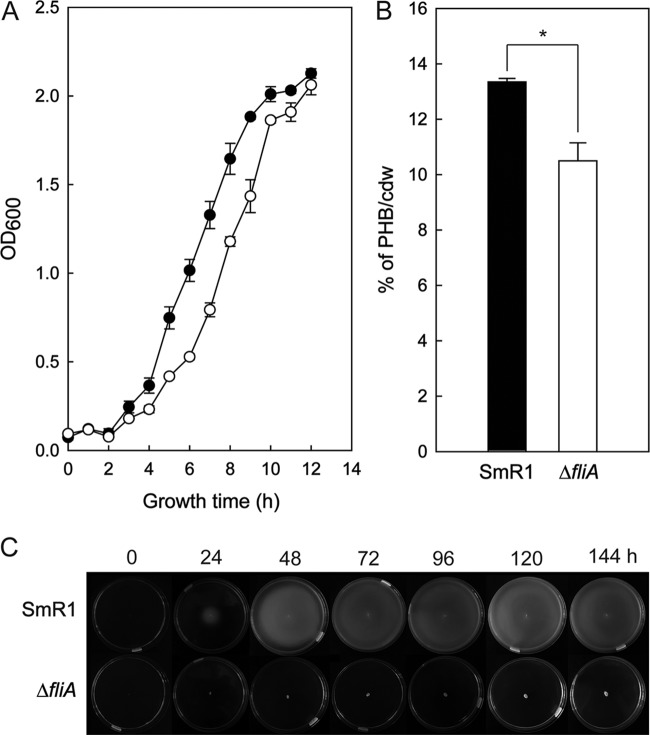
Growth, PHB accumulation, and motility profiles of H. seropedicae SmR1 and the Δ*fliA* mutant. (A) Bacteria were grown in NFbHPN-malate medium with 20 mM ammonium chloride and 37 mM dl-malate at 30°C and 120 rpm (orbital shaking). Bacterial growth was measured in function of the OD_600_ from three independent cultures. (B) PHB contents were measured by whole-cell methanolysis and gas chromatography from three independent samples of each strain when they reached an OD_600_ of 1.0 cultivated under the same conditions as for panel A. The quantity of accumulated PHB was normalized by the cell dry weight of lyophilized bacterial cultures used in the experiment. (C) Soft agar motility phenotype of Herbaspirillum seropedicae SmR1 and the Δ*fliA* mutant. The photographs are representative of four independent experiments. Overnight cultures were inoculated in the center of the plates containing NFbHPN-malate semisolid agar (0.25%, m/v) with 20 mM ammonium chloride and 37 mM dl-malate. The plates were incubated at 30°C and photographed every 24 h. Strain SmR1 and the Δ*fliA* mutant showed statistical significance against the other strains employed in the experiment (*, *P* value ≤ 0.01 [0.0098]) by the one-way ANOVA, *post hoc* Tukey test (*P* < 0.05).

As a control in our experiments, we also inoculated plants with the H. seropedicae Δ*fliA* mutant. In the genome of H. seropedicae SmR1, the Hsero_2029 gene was identified as a *fliA* homolog by amino acid sequence alignment against FliA (locus tag b1922; 49% identity) from Escherichia coli MG1655 (Fig. S2). *fliA* encodes the alternative sigma factor σ28 of the RNA polymerase that is responsible for initiation of transcription of several genes involved in motility and flagellar synthesis (45). The Δ*fliA* mutant of H. seropedicae is nonmotile on 0.25% (mass/volume [m/v]) agar and produces 20% less PHB than the parental strain SmR1, although the growths of the strains were similar (Fig. 5). The Δ*fliA* mutant was unable to colonize S. viridis plants endophytically (Fig. 4B). It is worth noting that the Δ*fliA* mutant was also defective in plant growth promotion (Fig. 1), but it is unclear whether this can be directly attributed to the lack of endophytic colonization by this strain.

### Monitoring PHB gene expression in H. seropedicae SmR1 colonizing Setaria viridis roots.

The data above suggest that PHB production by H. seropedicae associated with plant roots is important for the organism’s ability to promote plant growth, although it does not significantly impact total root colonization. However, this conclusion assumes that PHB is produced by bacteria either in the rhizosphere or on the rhizoplane. To demonstrate such production, as well as the capacity to utilize PHB in association with plant roots, we constructed H. seropedicae strains expressing *gfp* as a reporter under the control of the endogenous promoters of the genes *phaC1*, *phaZ1*, and *phaZ2*. S. viridis roots were inoculated with each of these strains, and *gfp* expression was monitored over time (i.e., 1 to 15 days postinoculation).

**FIG 6:**
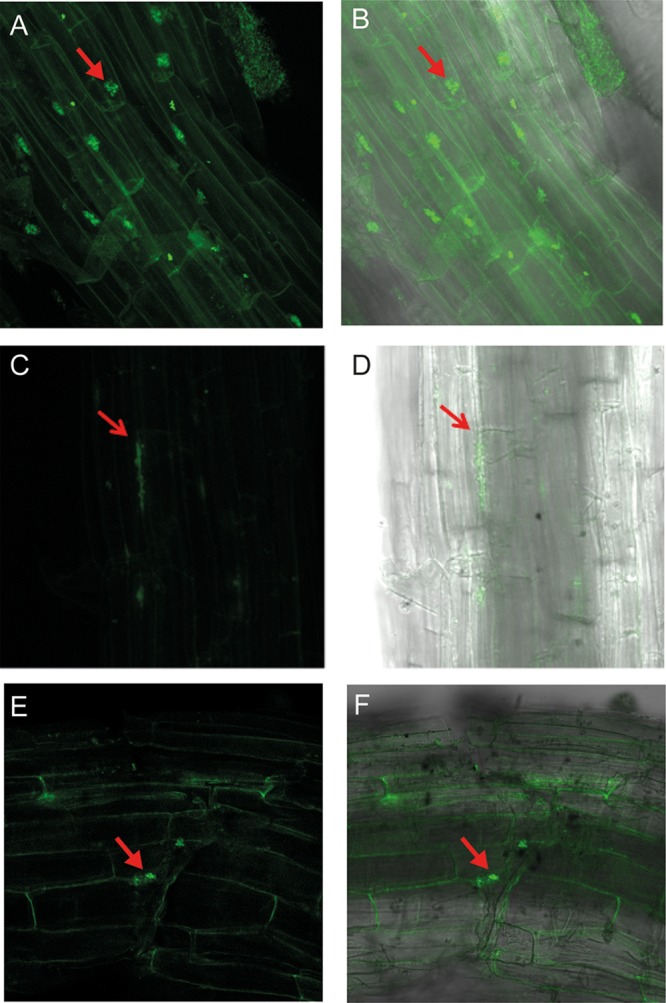
GFP expression from *phaC1*, *phaZ1*, and *phaZ2* promoter-*gfp* fusion in H. seropedicae SmR1 colonizing the S. viridis A10.1 root surface. (A and B) GFP expression from a P*phaC1-gfp* fusion in roots of 4-day-old plants. (C and D) GFP expression from a P*phaZ1-gfp* fusion in roots of 10-day-old plants. (E and F) GFP expression from a P*phaZ2-gfp* fusion in roots of 10-day-old plants. All the plants were washed and processed for the confocal microscopy analysis. Arrows indicate green fluorescent colonies of H. seropedicae. (A, C, and E) Confocal GFP fluorescence image; (B, D, and F) merged images of confocal fluorescence images and bright-field images.

As shown in Fig. 6A and B, expression of green fluorescent protein (GFP) in H. seropedicae cells colonizing S. viridis roots could be seen with the P*phaC1-gfp* fusion 4 days after inoculation. The images show bacteria forming microcolonies localized in the intercellular spaces of S. viridis root tissue. Similarly, significant expression by the strains expressing the P*phaZ1-gfp* or P*phaZ2-gfp* fusion was detectable 10 days after inoculation (Fig. 6C to F).

Interestingly, *gfp* expression by the P*phaC1-gfp* fusion was not detectable beyond 4 days after inoculation, while expression by the P*phaZ1-gfp* and P*phaZ2-gfp* fusions was only detectable 10 days after inoculation. The most straightforward explanation for these results is that PHB is synthesized early during bacterial colonization and, under the plant growth conditions used in our study, is mobilized much later, presumably to allow the bacteria to utilize the stored carbon. Uninoculated S. viridis roots showed no green fluorescence intensity (Fig. S3).

## DISCUSSION

Previous studies showed that many PHB-producing bacteria colonize plant roots and promote growth. A good example is Azospirillum brasilense, which was shown to promote plant growth in field experiments with maize and wheat in South America (46, 47). Okon and Itzigsohn (26) suggested that bacteria which produce and store PHB might improve cell division, survival, and stress tolerance due to energy release through PHB reserve mobilization when required.

Previous work demonstrated that H. seropedicae SmR1 could colonize S. viridis A10.1, resulting in a significant enhancement of growth (38). The current study examined whether PHB synthesis in this system was required for either root colonization or plant growth promotion. The wild-type strain, strains producing significantly less PHB, and strains totally lacking PHB exhibited similar levels of root colonization. However, strains with those mutations that either reduced PHB synthesis or eliminated it showed a significant reduction in their ability to promote plant growth. Among the mutants with a reduced quantity of PHB, the Δ*phaR* mutant presented the worst root development parameters after 25 days of inoculation. Interestingly, the deletion of *phaR* also reduced PHB production in Rhizobium etli (48) and Bradyrhizobium diazoefficiens (16). However, the Δ*phaR* mutant of Bradyrhizobium diazoefficiens was more competitive than the wild type for nodulation of soybean plants and enhanced plant dry matter. Our results compared to those of Quelas et al. (16) demonstrate that the phenotypes generated by disruption of PHB metabolism can differ depending on the type of bacteria and the environment that they are colonizing. Even for the insect host, the bean bug Riptortus pedestris, the deletion of the *phaR* gene in a *Riptortus* symbiont *Burkholderia* sp. reduced PHB levels *in vitro* and *in vivo* when colonizing the midgut of the insect (49). As a consequence, the body weights of male and female insects fed with the Δ*phaR* mutant were decreased compared to those fed with the wild-type *Burkholderia* sp. (49).

In order to verify the influence of PHB mobilization during root colonization, we tested the colonization and plant growth response upon inoculation with the Δ*phaZ1* Δ*phaZ2* mutant. This mutant did not survive 30 min of heat shock at 45°C, due to a lack of the PHA depolymerases and the consequent inability to mobilize carbon from PHB granules (data not shown). Similar results were obtained by Ruiz and coworkers when wild-type Pseudomonas oleovorans and a *phaZ* mutant strain were subjected to elevated temperature (47°C) (20). While the *phaZ1* and *phaZ2* deletion did not affect colonization, it did significantly reduce the ability of H. seropedicae to stimulate S. viridis growth. This result corroborates previous work suggesting that PHB mobilization is a relevant feature to increase the root surface area and uptake of minerals from soil (50–53).

A role for PHB in bacterial growth promotion assumes that PHB synthesis/depolymerization occurs in the root rhizosphere and/or in the rhizoplane. Consistent with this assumption, we demonstrated GFP expression on the plant root surface using H. seropedicae strains expressing *phaC1*, *phaZ1*, or *phaZ2* promoter-*gfp* fusions.

Our results are consistent with those of Pankievicz et al. (32), which showed a 1.9-fold increase of *phaC1* mRNA levels in H. seropedicae SmR1 colonizing wheat roots. Nevertheless, we observed that the genes were expressed at different times during root colonization. These data suggest that PHB synthesis and accumulation by H. seropedicae occur early during root colonization, perhaps reflecting an abundance of root exudates, containing predominately organic acids and sugars (54, 55), which can sustain the synthesis and accumulation of PHB. The expression of the P*phaZ1-gfp* and P*phaZ2-gfp* fusions 10 days after inoculation suggests that PHB mobilization occurs only after extensive bacterial root colonization, when root-derived carbon may be less available. These results are consistent with those reported by Lodwig and coworkers (28), who showed that bacterial accumulation of PHB promoted plant infection and increased bacterial differentiation during colonization. Furthermore, Koskimäki et al. demonstrated that Methylobacterium extorquens produced 3-hydroxybutyrate oligomers during colonization of Pinus sylvestris roots (22).

In a number of bacteria, PHB synthesis is known to increase under low-oxygen conditions. Azotobacter beijerinckii was reported to produce PHB as an adaptation to the low-oxygen environment on legume roots (40). Indeed, PHB oligomers have potent antioxidant activity against hydroxyl radicals and, hence, may protect sensitive cellular functions under microaerobic growth. Consistent with such a role, the H. seropedicae Δ*phaC1* mutant strain produces a higher reactive oxygen species (ROS) level (37).

Our results show that only the parental SmR1 strain and the Δ*phaP2* mutant, which produce a significant amount of PHB granules inside the cell, grew normally under microaerobic conditions. In contrast, the other mutants affected in PHB metabolism grew significantly more slowly under a low-oxygen regimen. Hence, there appears to be a correlation among the ability to produce PHB, grow normally under low-oxygen conditions, and promote plant growth. Unfortunately, the detailed mechanism by which PHB may contribute to bacterial plant growth promotion remains unclear. However, the results with the *phaZ* mutants argue that the ability to depolymerize the PHB polymer, presumably to harvest the stored carbon, is a critical aspect.

In conclusion, our results demonstrate that PHB metabolism contributes to the plant growth promotion ability of H. seropedicae, although it did not significantly affect epiphytic or endophytic colonization by the bacteria. Thus, the PHB cycle likely ensures the proper metabolic state to allow the bacteria to express traits that promote plant growth.

## MATERIALS AND METHODS

### Bacterial strains and growth conditions.

Herbaspirillum seropedicae SmR1 parental (56) and mutant strains were cultivated in NFbHPN-malate medium containing 37 mM dl-malic acid, 20 mM phosphate, and 20 mM NH_4_Cl (57) and streptomycin (80 µg/ml) at 30°C and 120 rpm. To grow the bacteria under low-oxygen conditions, we employed 30-ml glass flasks filled with 25 ml of medium. After inoculation, the flasks were closed with a rubber cap and incubated in an orbital shaker at 100 rpm at 30°C. Samples were taken every 2 h and growth measured by determination of the optical density at 600 nm (OD_600_). Several H. seropedicae mutants defective in PHB metabolism were used. Escherichia coli EC100 (Epicentre, Madison, WI) and S17-1 (58) were used for cloning and conjugation procedures. They were grown in LB medium (59) at 37°C at 160 rpm. All the strains and plasmids used in this work are described in Table 1.

**TABLE 1:** Bacterial strains and plasmids used in this work

Strain or plasmid	Relevant characteristics	Source or reference
E. coli strains		
EC100	Cloning and plasmid maintenance	Epicentre
S17-1	Conjugation strain	58
H. seropedicae strains		
SmR1	Parental strain; Nif^+^ Sm^r^ PHB^+^	56
Δ*phaP1*	Chromosomal deletion of *phaP1*, reduction in PHB accumulation	36
Δ*phaP2*	Chromosomal deletion of *phaP2*	36
Δ*phaP1* Δ*phaP2*	Chromosomal deletion of *phaP1* and *phaP2*, PHB^−^ (PHB granules are not accumulated)	13
Δ*phaC*1	Chromosomal deletion of *phaC1*, PHB^−^ (PHB is not synthetized), oxidative stress sensitive	36
Δ*phaZ1*	Chromosomal deletion of *phaZ1*	This work
Δ*phaZ2*	Chromosomal deletion of *phaZ2*	This work
Δ*phaZ1* Δ*phaZ2*	Chromosomal deletion of *phaZ1* and *phaZ2*; PHB^+^; unable to degrade PHB	This work
Δ*phaR*	Chromosomal deletion of *phaR*, reduction in PHB accumulation	This work
Δ*fliA*	Chromosomal deletion of *fliA*; PHB^+^; nonmotile	This work
Plasmids		
pK18*mobsacB*	Suicide vector; Km^r^ *sacB*; mobilizable plasmid	60
pK18mobΔ*fliA*	pK18*mobsacB* harboring Δ*fliA* overlapping PCR product	This work
pK18mobΔ*phaZ1*	pK18*mobsacB* harboring Δ*phaZ1* overlapping PCR product	This work
pK18mobΔ*phaZ2*	pK18*mobsacB* harboring Δ*phaZ2* overlapping PCR product	This work
pK18mobΔ*phaR*	pK18*mobsacB* harboring Δ*phaZ1* Δ*phaZ2* overlapping PCR product	This work
pBBR1MCS-3	Broad-host-range vector	66
pMJP01	pBBR1MCS-3 derivative harboring a 1,151-bp PCR product containing *phaR* and its upstream regulatory region	This work
pGWB-4 (e-GFP)	Construction of transcriptional fusions with the eGFP^*a*^ as a gene reporter	65
pPHAC1GFP	eGFP under the control of the *phaC1* promoter of H. seropedicae SmR1	This work
pPHAZ1GFP	eGFP under the control of the *phaZ1* promoter of H. seropedicae SmR1	This work
pPHAZ2GFP	eGFP under the control of the *phaZ2* promoter of H. seropedicae SmR1	This work

a eGFP, enhanced GFP.

### Construction of H. seropedicae SmR1 mutants by gene deletion.

The in-frame markerless deletion of *fliA* (Hsero_2029; GenBank accession number ADJ63529) was obtained by cloning upstream and downstream fragments of the gene into the nonreplicating plasmid pK18*mobsacB*, which carries a kanamycin resistance cassette along with *sacB*, which confers sucrose sensitivity (60). Briefly, a 527-bp upstream *fliA* fragment, including its first 30 nucleotides, was amplified by PCR with primers Fw_*fliA*_UP and Rev_*fliA_*UP (Table 2). To amplify a 530-bp downstream *fliA* fragment, including its last 33 nucleotides, PCR was done with primers Fw_*fliA*_DOWN and Rev_*fliA_*DOWN. Overlapping PCR fused the PCR products, and the resulting Δ*fliA* product was cloned into pBlueScript II KS+ (61). The entire construction was digested with EcoRI and HindIII to ligate the Δ*fliA* fragment into pK18*mobsacB* digested with the same enzymes, yielding pK18Δ*fliA*. E. coli S17-1 was transformed with pK18Δ*fliA* and the plasmid conjugated to H. seropedicae SmR1 using biparental mating. Single recombinants were selected on NFbHPN-malate agar containing streptomycin (80 µg·ml^−1^), nalidixic acid (5 µg·ml^−1^), and kanamycin (500 µg·ml^−1^). A single-recombinant colony was collected in 3 ml of NFbHPN-malate and cultivated overnight without antibiotics. The culture was serially diluted and plated on NFbHPN-malate agar containing 10% (wt/vol) sucrose. Colonies that grew on sucrose were screened for deletion by PCR using the primers Fw_*fliA*_UP and *fliA*_internal, which anneals in the deleted region. The presence of an amplicon of 862 bp indicates the restitution of the wild-type *fliA*, while no amplification is expected in a Δ*fliA* mutant. The same procedure was applied to delete *phaR* (Hsero_2997; GenBank accession number ADJ64485), *phaZ1* (Hsero_1622; GenBank accession number ADJ63135), and *phaZ2* (Hsero_0639; GenBank accession number ADJ62158) in H. seropedicae SmR1. To construct the Δ*phaZ1* Δ*phaZ2* mutant, the Δ*phaZ1* mutant was conjugated with pK18Δ*phaZ2* and colonies were screened for double deletion.

**TABLE 2:** Primers used in this study^*a*^

Primer name	Sequence (5→3)
Fw_*fliA*_UP	GTGAATTCGGCCAACTGGCGGAGATCTT
Rev_*fliA*_UP	GGATCCCTTAGGTCGTCTCTACTTGCCTTTTTTTCCCTTGAC
Fw_*fliA*_DOWN	TAGAGACGACCTAAGGGATCCCTGCGCGAGCATTCCTGGAGCG
Rev_*fliA*_DOWN	GGTTAAGCTTGTCGAAAAACTGCGCCAGGA
Fw_*phaR*_UP	GAGGATCCCGTGACCGTCAACACCGTCT
Rev_*phaR*_UP	AGATCTCTTAGGTCGTCTCTATGCAGTAGTCATCTGAAGTCCAGTC
Fw_*phaR*_DOWN	TAGAGACGACCTAAGAGATCTATGTTCGGCACCTTCCCC
Rev_*phaR*_DOWN	GTTCTGCAGTTGCCGCGATTCATGGTGG
Fw_*phaZ1*_UP	TCGGATCCCAAGCAACTGAAGGTGAT
Rev_*phaZ1*_UP	GATATCCTTAGGTCGTCTCTACTTGGCTGAGGTCTCCGC
Fw_*phaZ1*_DOWN	TAGAGACGACCTAAGGATATCTACGGCATCTTCTCGGGC
Rev_*phaZ1*_DOWN	TCTCTAGATGCTGATGCAATCGACCG
Fw_*phaZ2*_UP	TCGGATCCCCATACCAGTACGTCGCCA
Rev_*phaZ2*_UP	AGATCTCTTAGGTCGTCTCTACAGCGCCTGCGCCATCATC
Fw_*phaZ2*_DOWN	TAGAGACGACCTAAGAGATCTCATTACGGCGTCTTCAAC
Rev_*phaZ2*_DOWN	TTCTGCAGTGAATTCGGTGGTCTTCT
Fw_*phaR*c_Hs	TGTCTGCAGCGGTTCGGACTTCTCCCTCA
Rev_*phaR*c_Hs	TGTGAGCTCTGCACTTCCGGAGCCTTTACCTGC
Fw_phaC1_prom	GGGGACAAGTTTGTACAAAAAAGCAGGCTACTTTTCGGCGCTGCTTCTTC
Rev_phaC1_prom	GGGGACCACTTTGTACAAGAAAGCTGGGTCCAGAACTTCGGATCGGACAT
Fw_phaZ1_prom	GGGGACAAGTTTGTACAAAAAAGCAGGCTAAGGCAAGAACATCGTCATCT
Rev_phaZ1_prom	GGGGACCACTTTGTACAAGAAAGCTGGGT TTCATGCAGTTGATAAAGCAT
Fw_phaZ2_prom	GGGGACAAGTTTGTACAAAAAAGCAGGCTCGATGTCCTCCGGTTCCTTCT
Rev_phaZ2_prom	GGGGACCACTTTGTACAAGAAAGCTGGGTGAGCTGATAGGTAGGGATCAT

a The primers were generated in this study.

### Quantification of PHB by GC-FID.

PHB was quantified by methanolysis of 5 to 10 mg of lyophilized bacteria and GC-FID (gas chromatography coupled to the flame-ionization detector) analyses following the protocol previously reported (62). Amounts of PHB in each sample were expressed as percent PHB per cell (dry weight) (percent mass/mass).

### Soft agar motility assay.

The soft agar motility of H. seropedicae and the Δ*fliA* mutant was evaluated on NFbHPN-malate semisolid agar (0.25%, m/v) plates. Fresh colonies of Herbaspirillum seropedicae SmR1 and the Δ*fliA* mutant were grown in NFbHPN-malate medium with streptomycin (80 µg ml^−1^) under 30°C at 120 rpm (57). This culture was used to inoculate a new NFbHPN-malate medium. After 16 h under 30°C at 120 rpm, a saturated culture was used to inoculate the center of the semisolid agar plates with streptomycin (80 µg ml^−1^). The plates were incubated at 30°C and photographed every 24 h to follow the formation of halos in semisolid medium.

### Sterilization and germination of Setaria viridis A10.1 seeds.

Seeds of S. viridis were surface sterilized with a 1% sodium hypochlorite plus 0.1% (vol/vol) Tween 20 solution for 3 min, followed by three rinses with distilled sterile water. Sterilized seeds were plated onto modified Hoagland’s nutrient solution (63) containing 1% (vol/vol) phytagel. The solution was autoclaved for 30 min at 121°C. Plates were placed horizontally in the dark for 1 day, followed by 2 days in the light at 30°C.

### Inoculation and growth of Setaria viridis A10.1 plants.

Three-day-old seedlings of S. viridis were inoculated with H. seropedicae SmR1 or one of the mutant strains to be evaluated. The bacterial cultures were grown in the NFbHPN-malate medium as described above until an OD_600_ of 1.0 (10^8^ cells ml^−1^) was reached. The culture was washed twice with sterile 0.9% saline solution and diluted to 1 × 10^7^ cells ml^−1^ before inoculation. The seedlings were inoculated for 30 min with 1 ml of this bacterial suspension per plantlet and then transferred to pots containing a mixture of sterile Turface and vermiculite in a proportion of 3:1 (wt/wt). In total, 3 groups of 30 plantlets were inoculated on 3 consecutive days with fresh bacterial culture. Plants were grown in the greenhouse at 30°C with a 16-h light/8-h darkness cycle for 25 days. Plants were watered twice a week with Hoagland’s solution supplemented with 0.5 mM potassium nitrate (KNO_3_).

### Quantification of bacterial colonization.

To verify the ability of H. seropedicae SmR1 and mutant strains to colonize S. viridis roots, a colonization assay was performed using plants harvested 25 days postinoculation. To quantify the epiphytic colonization, roots were vortexed in 1 ml of saline solution (0.9% sodium chloride) for 1 min. The bacterial suspension was diluted and the CFU number counted as described below. To quantify the endophytic colonization, roots were surface sterilized with 70% ethanol for 1 min followed by 1% sodium hypochlorite for 1 min and then washed thrice using sterilized distilled water. The sterile roots were then ground in 1 ml of saline solution. The root extract was 10-fold serially diluted, and 10 µl of each suspension was pipetted onto NFbHPN-malate medium containing 80 µg ml^−1^ of streptomycin. The CFU number was counted after 2 days of incubation at 30°C. The number of CFU was normalized by the dry weight of roots in grams.

### Plant growth promotion analysis.

S. viridis plants, 25 days postinoculation, were harvested by carefully removing the soil and washing the roots briefly with distilled water before growth parameter measurements (i.e., root and shoot length, lateral root number, and root and shoot fresh and dry weight). The root length and the number of lateral roots were analyzed using WinRHIZO 2002c software (Regent Instrument Inc., Quebec City, Canada), the shoot length was measured using a ruler, and the fresh shoot and root weights were determined by weighing on an analytical balance.

### Statistical analysis.

Statistical analyses were performed by one-way analysis of variance (ANOVA) with *post hoc* Tukey honestly significant difference (HSD; *P* ≤ 0.05) at the 95% confidence level by R package software version 3.5.1 (64). The mean values for SmR1 (parental strain) were compared to the uninoculated and mutant mean values to determine the statistical difference. The number of plants varied in each treatment due to variability in plant growth and survival.

### Expression analysis of transcriptional fusions of *pha* promoters with *gfp* reporter in H. seropedicae SmR1.

The promoter regions upstream of the *phaC1* (P*phaC1*; locus tag Hsero_2999), *phaZ1* (P*phaZ1*; Hsero_1622), and *phaZ2* (P*phaZ2*; Hsero_0639) genes were amplified from H. seropedicae SmR1 genomic DNA by PCR and cloned into Gateway pDONR/Zeo. The clones were obtained by BP clonase *in vitro* recombination overnight followed by proteinase K treatment for 10 min at 37°C and transformed in E. coli EC100; then the colonies were grown and a PCR was done to check and confirm the clones. The validity of each cloned region was confirmed by sequencing. LR cloning was done as previously described to BP clonase, though using LR clonase in a recombination reaction to transfer the fusions to the pGWB-4 (65). Following confirmation by PCR, each construct was transformed into E. coli S17-1 before mating with H. seropedicae SmR1.

### Analysis of bacterial *pha* gene expression on S. viridis roots by confocal microscopy.

In order to determine the expression of PHB genes during S. viridis root colonization, 3-day-old seedlings were inoculated with H. seropedicae SmR1 harboring the *pha* gene promoter-*gfp* transcriptional fusions. Dissected root fragments from control and inoculated plants were placed on a slide in a drop of water and covered with a glass coverslip to be observed under a fluorescence microscope (IX70 inverted microscope; Olympus, Melville, NY). For confocal microscopy, the roots were prepared as described above and observed using a Zeiss (Oberkochen, Germany) LSM 510 META laser scanning confocal microscope equipped with 488-nm Ar and 543-nm He-Ne lasers to detect green fluorescence emitted by GFP-tagged H. seropedicae (excitation at 488 nm and detection at 500 to 550 nm).

Roots were observed 1, 4, 7, 10, and 15 days after inoculation with H. seropedicae SmR1 carrying the *pha* gene promoter-*gfp* fusions. The green fluorescence images from GFP-tagged bacteria and the transmitted images (bright-field mode) of the identical image were overlaid. All composite images were produced using LSM Image Browser 4.0 software (Carl Zeiss Microimaging, Oberkochen, Germany). Additional images were obtained using a Nikon (Melville, NY) Eclipse Ti inverted laser scanning confocal microscope equipped with an ion laser to detect green fluorescence.

## Supplementary Material

Supplemental file 1
