# Supplementary material for: Importance of Poly-3-Hydroxybutyrate Metabolism to the Ability of Herbaspirillum seropedicae To Promote Plant Growth
Source: Appl Environ Microbiol. 2019 Mar 6;85(6):e02586-18. doi: 10.1128/AEM.02586-18 (PMC6414382; doi:10.1128/AEM.02586-18)
Supplement: Supplemental file 1 [file AEM.02586-18-s0001.pdf]

SUPPLEMENTARY DATA

FIG. S1. Multiple alignments of protein sequences of (A) PhaZ1 from *H. seropedicae* SmR1 (Hsero_1622) against PhaZ1a1 from *R. eutropha* H16 (A1150) and (B) PhaZ2 from *H. seropedicae* SmR1 (Hsero_0639) against PhaZ2a2 from *R. eutropha* (A2862) were carried out using Clustal Omega and the default parameters (1). The asterisks (*) indicate fully conserved residues, while colons (:) indicate conservation in residues of strongly similar properties and points (.) indicate conservation in residues of weakly similar properties.

A

```
PhaZ1        MLYQLHELNRAFLNPMMQWAETSAKLFSDPVSPLAHTPFSQRIAAGYELLYRLSKEYEKP    60
PhaZ1a1      MLYQLHEFQRSILHPLTAWAQATAKTFTNPLSPLSLVPGAPRLAAGYELLYRLGKEYEKP    60
             *******::*::*:*:  **:::** *::*:***: .* : *:**********.******

PhaZ1        QFNIDAVPVDGKSVGIVEEVVEEKPFCRLIHFRKDLSARQAT--ALKQPTVLVVAPLSGH    118
PhaZ1a1      AFDIKSVRSNGRDIPIVEQTVLEKPFCKLVRFKRYADDPETIKLLKDEPVVLVAAPLSGH    120
              *:*.:*  :*:.: ***:.* *****:*::*::  .  ::     .:*.***.******

PhaZ1        HSTLLRETVRALLQEHDVYITDWTDARMVPVEQGAFHLHDYVYYVQDFIRTLGP-DVHVI    177
PhaZ1a1      HATLLRDTVRTLLQDHKVYVTDWIDARMVPVEEGAFHLSDYIYYIQEFIRHIGAENLHVI    180
             *:****:***:***:*.**:*** ********:***** **:**:*:*** :*  ::***

PhaZ1        SVCQPTVPVLAAISLMASANDPMLPKSMTMMGGPIDARKSPTAVNDLATEKPYSWFENTV    237
PhaZ1a1      SVCQPTVPVLAAISLMASAGE-KTPRTMTMMGGPIDARKSPTAVNSLATNKSFEWFENNV    239
             *******************.:   *::******************.***:* :.****.*

PhaZ1        IYSVPANYPGFGRKVYPGFLQHAGFVAMNPRRHAQSHWDFYMHLRDGDDASAEEHRKFYD    297
PhaZ1a1      IYTVPANYPGHGRRVYPGFLQHAGFVAMNPDRHLSSHYDFYLSLVEGDADDAEAHVRFYD    299
             **:*******.**:**************** ** .**:***: * :**  .** * :***

PhaZ1        EYNAVLDMPAEFYLETIKVVFQDFNLARGTWEIEGQLVRPQDIKSVALFTIEGELDDISG    357
PhaZ1a1      EYNAVLDMAAEYYLDTIREVFQEFRLANGTWAIDGNPVRPQDIKSTALMTVEGELDDISG    359
             ******** **:**:**: ***:*.**.*** *:*: ********.**:*:*********

PhaZ1        SGQTQAAQELCSSIPKARKQHFTVPKAGHYGIFSGRRWREIVCPKIGEFIRANA------    411
PhaZ1a1      AGQTAAAHDLCAGIPKIRKQHLNAAHCGHYGIFSGRRWREEIYPQLRDFIRKYHQASATR    419
             :*** **::**:.*** ****:.. :.************* : *:: :***
```

B

```
PhaZ2        MIPTYQLYQNYADATDPLRACARMMAQALGATWPGIPVHPYWRKMASACEVFARTQLTHA    60
PhaZ2a2      --MLYHAYQIYADMILPACTLAELAAATLAA-NPRSGGFDAVPRLRAACELIALVRLTHH    57
                 *: ** ***   *  : *.: * :*.*  *    .    :: :***::* .:***

PhaZ2        RPPFGIATVEEEGCTISVHEEEIHATPFCGLLHFRKDSATVQPKVLVVAPMSGHFATLLR    120
PhaZ2a2      RPAFGIDHATVGGQPVPVTEEVVARTPFCSLLHFRRHGIVGQPRVLLVAPMSGHFATLLR    117
             ** ***   .  *  : * ** :  ****.*****:.. . **:**:*************

PhaZ2        GTVRTLLRDHDVYITDWRNARDVATAHGRFGLDEYVSHIIDFLGVLGPGAHLLAVCQPTV    180
PhaZ2a2      GTVQTMLADHDVYLTDWHNPRDIPLLAGRFGFDEFVQHLIGFLQTLGGGTHLVAICQPAV    177
             ***:*:* *****:***:* **:    ****:**:*.*:*.** .** *:**:*:***:*

PhaZ2        AALTAAAVMAADGHPAQPRSMTLMAGPIDTRVNPTAVNALAKSKPIAWFEKNMISTVPAR    240
PhaZ2a2      AALAAAALMAEDGDPAQPPSLTLMAGPIDARVNPTKVNALAMSQPLEWFERTLIGMVPLR    237
             ***:***:** **.**** *:********:***** ***** *:*: ***:.:*. ** *

PhaZ2        HAGAGRRVYPGFVQLAAFMNMNLSRHVEAFGKLYHHLVDGEHARADQIKDFYEEYFAMAD    300
PhaZ2a2      FAGAMRRVYPGHVQLLAFMSMNPERHEQALRELYALRERGEHDKADAIRDFYIEYFATMD    297
             .*** ******.*** ***.** .** :*: :**     *** :** *:*** ****  *

PhaZ2        LPAEFYLETVRTVFQEHALPLGKLSYAGRPVEPRAIRRTALFTIEGEKDDICAVGQTLAA    360
PhaZ2a2      LTAEFYLETVSLVFQRFLLAQGLLDVSGRRVRTRAIHRTALLTVEGERDDICAIGQTVAA    357
             * ********  ***.. *  * *. :** *. ***:****:*:***:*****:***:**

PhaZ2        QELCSGIRPYMRLHHVQTAVGHYGVFNGRRWDNEIYPRLRDFINMHHR   408
PhaZ2a2      QDLCSSLRPYMRMHHVQTGVGHYGVFNGRRWETQVYPLVRNTIYTSS-   404
             *:***.:*****:*****.************:.::** :*: *
```

FIG. S2. Multiple alignment of protein sequences of FliA from *H. seropedicae* SmR1 (Hsero_2029) against FliA from *E. coli* MG1655 (b1922) was carried out using Clustal Omega and the default parameters (1). The asterisks (*) indicate fully conserved residues, while colons (:) indicate conservation in residues of strongly similar properties and points (.) indicate conservation in residues of weakly similar properties.

```
FliA_Hs      ---MYNVKGKKGKNDLLEQHAPLVKKLAHQLKAKLPPSVEVDDLIQAGMMGLLDAVNRYE     57
FliA_Ec      MNSLYTAEGVMDKHSLWQRYVPLVRHEALRLQVRLPASVELDDLLQAGGIGLLNAVERYD     60
                :*..:*  .*:.* :::.***:: * :*:.:** ***:***:*** :***:**:**:

FliA_Hs      ETHGAQFETYAVQRIRGAMLDELRSSDWLPRSIRQNARKVEEAMQTLLQQLGRQPKEAEL     117
FliA_Ec      ALQGTAFTTYAVQRIRGAMLDELRSRDWVPRSVRRNAREVAQAIGQLEQELGRNATETEV     120
               :*: * ***************** **:***:*:***:* :*:  * *:***: .*:*:

FliA_Hs      AQHMQISLEDYQSLLSECGGHQLIYYEDFHDDDGGHEHFLDRHQTSDSDDPLQNLINGGF     177
FliA_Ec      AERLGIDIADYRQMLLDTNNSQLFSYDEWREEHGDSIELVTD--DHQRENPLQQLLDSNL     178
             *::: *.: **:.:* : .. **: *::::::.*.  .::      : ::***:*::..:

FliA_Hs      REAVVDSIKALPEREQILMALYYEQEMNLKEIGAVMGVSESRVCQLHSQAISRMRSYLRE     237
FliA_Ec      RQRVMEAIETLPEREKLVLTLYYQEELNLKEIGAVLEVGESRVSQLHSQAIKRLRTKLGK     238
             *: *:::*::*****:::::***::*:********: *.****.*******.*:*: * :

FliA_Hs      HSWSGVA   244
FliA_Ec      L------   239
```

FIG. S3. Confocal microscopy analysis of uninoculated *Seteria viridis* roots showing a low intrinsic green fluorescence. (A) Confocal GFP fluorescence image and (B) merged images of confocal fluorescence and bright field images

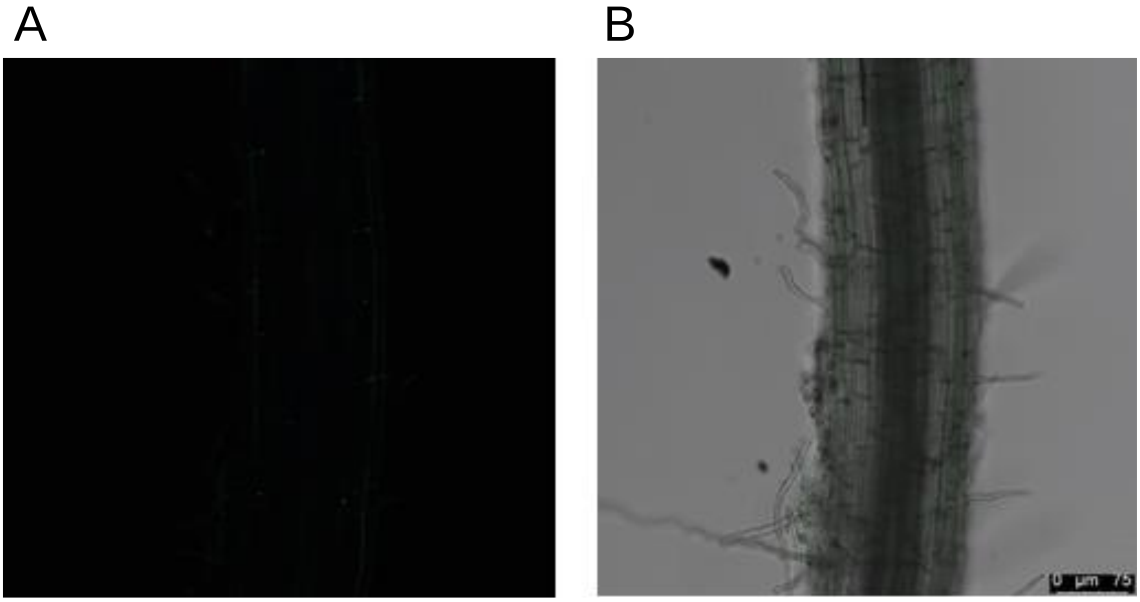

References

1. Sievers F, Wilm A, Dineen D, Gibson TJ, Karplus K, Li W, Lopez R, McWilliam H, Remmert M, Soding J, Thompson JD, Higgins DG. 2011. Fast, scalable generation of high-quality protein multiple sequence alignments using Clustal Omega. Mol Syst Biol 7:539.
